# Supplementary figures and images for: Dissection of Nidogen function in Drosophila reveals tissue-specific mechanisms of basement membrane assembly
Source: PLoS Genet. 2018 Sep 27;14(9):e1007483. doi: 10.1371/journal.pgen.1007483 (PMC6177204; doi:10.1371/journal.pgen.1007483)

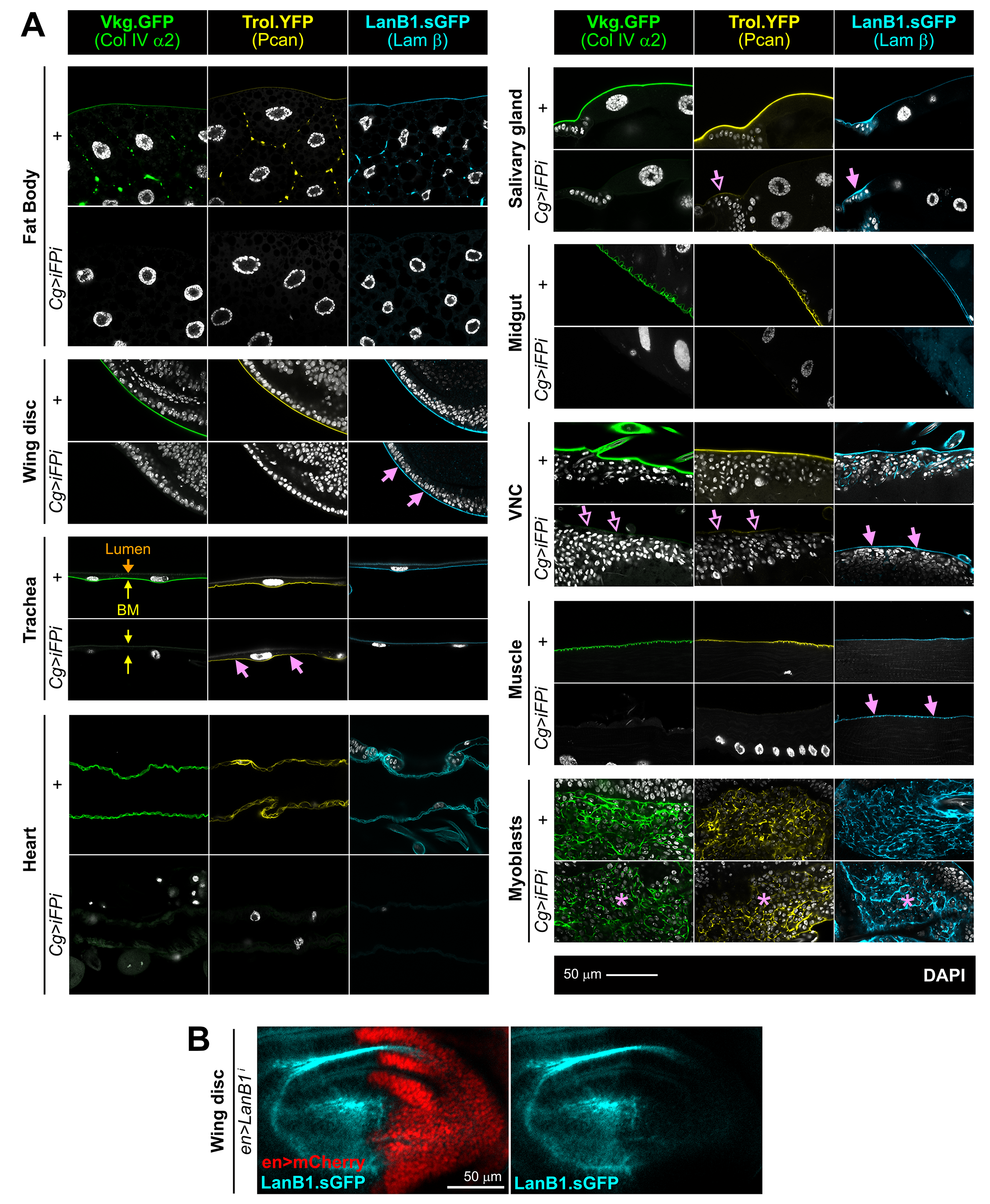

Supplement: S1 Fig — (A) Confocal images showing the localization of Collagen IV (Vkg.GFP, green), Perlecan (Trol.YFP, yellow) and Laminin (LanB1.sGFP, cyan) in different tissues of the 3rd instar larva. Images compare control tissues (+) with tissues from larvae where expression of the corresponding fluorescence protein fusion has been knocked down through Cg-GAL4-driven iGFPi (Cg>isGFPi). Disappearance of the corresponding signal from BMs is observed, with the exceptions indicated by hollowed arrows (partial reduction) and asterisks or filled arrows (no reduction). Nuclei stained with DAPI (white). (B) LanB1.sGFP signal (cyan) in the posterior compartment of the wing disc is reduced in en>LanB1i larva. Posterior compartment cells (en+) express mCherry (red). (TIFF) [file pgen.1007483.s001.tiff]

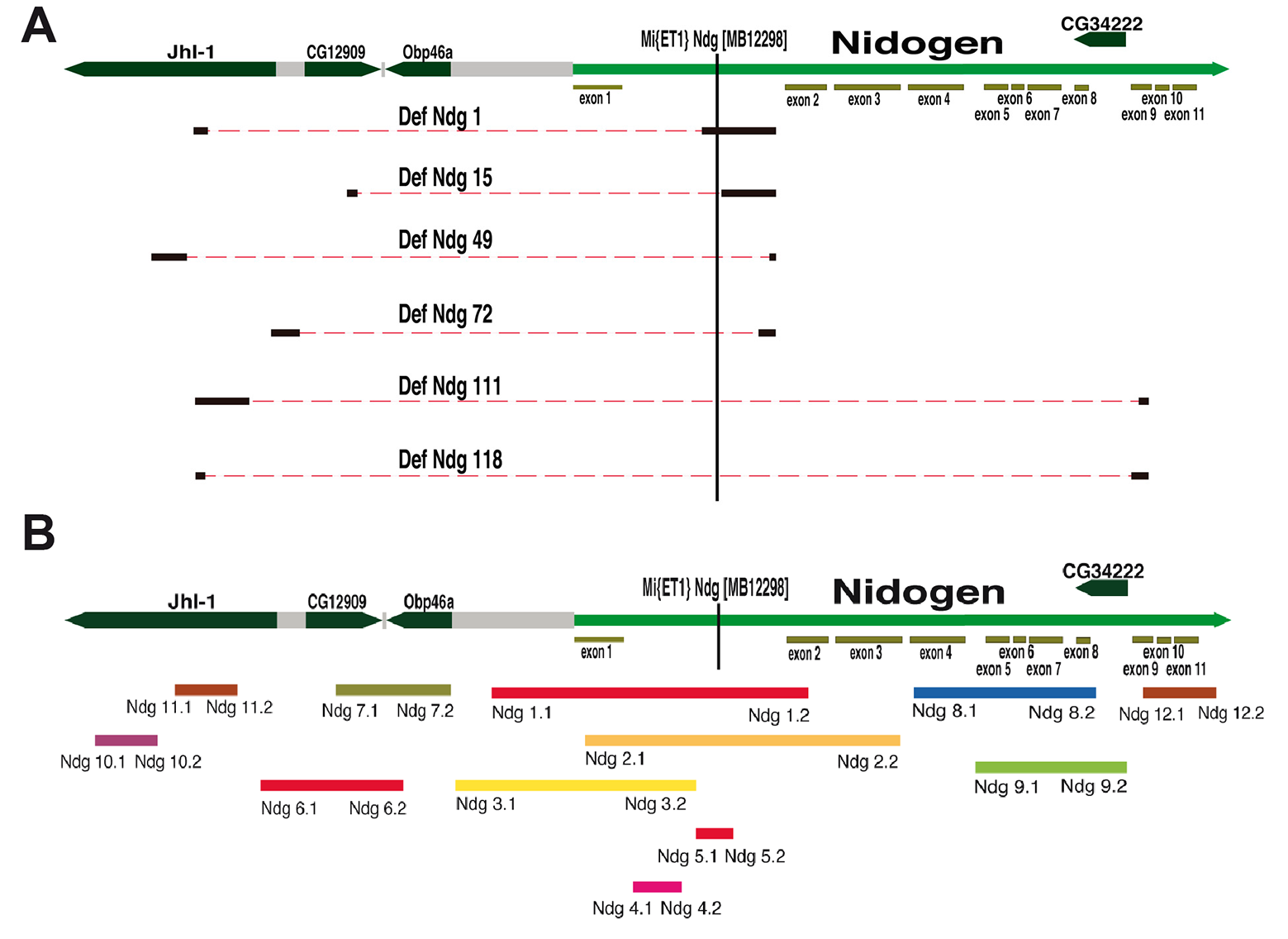

Supplement: S2 Fig — (A) Schematic representation of the deficiencies generated in the Ndg region by imprecise excision of the Mi{ET1}Ndg[MB12298] transposon (see Materials and Methods). (B) Colour-code diagram picture of the primer pairs used for molecular characterization of the deficiencies described in (A) (see Materials and Methods). (TIF) [file pgen.1007483.s002.tif]

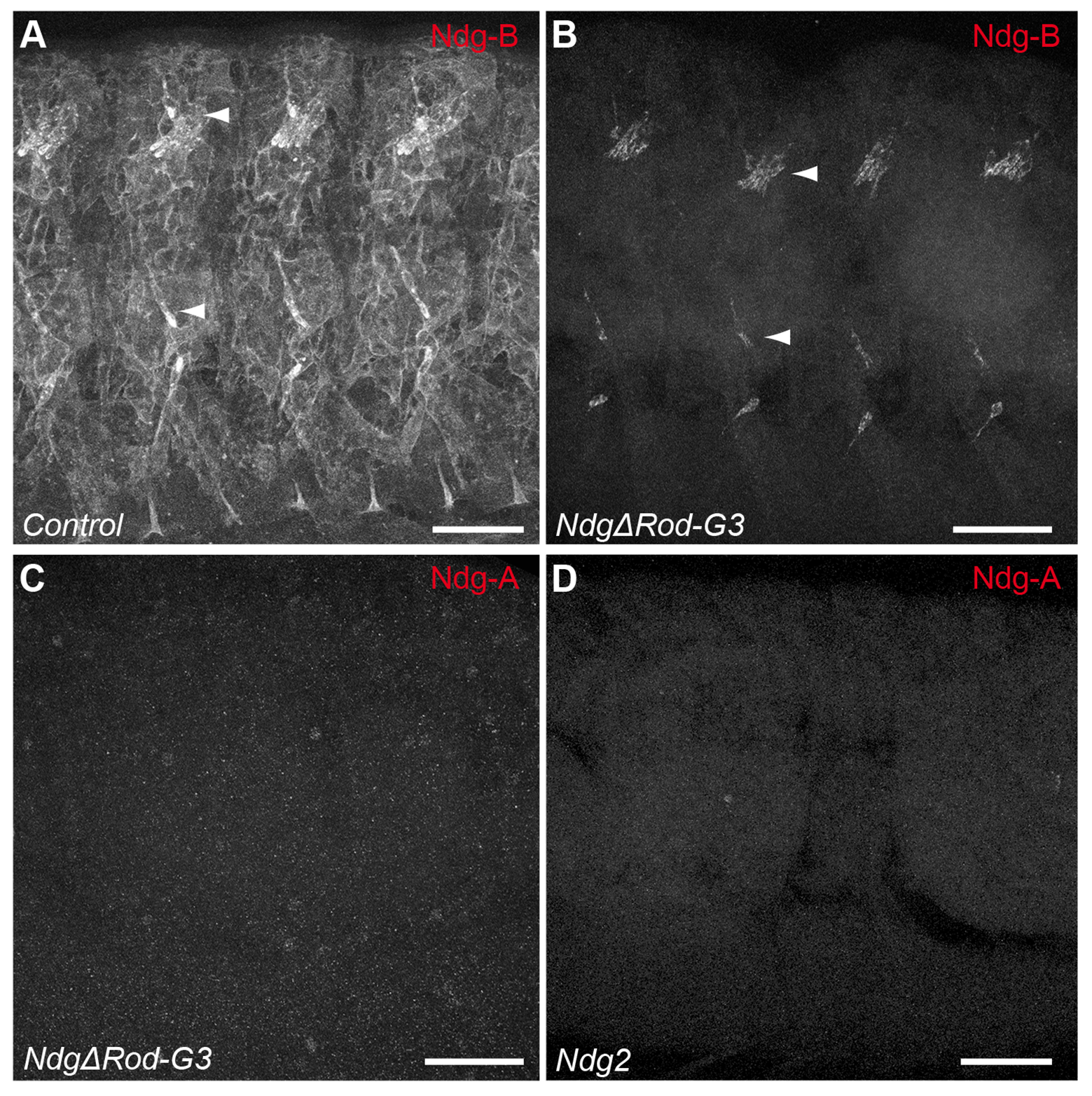

Supplement: S3 Fig — (A-D) Confocal images showing stage 16 embryos stained with two different anti-Ndg (red) antibodies. Images compare control embryos (A) with NdgΔRod-G3.1 embryos (B, C). (A-B) Stage 16 control (A) and NdgΔRod-G3.1 (B) mutant embryos stained with an anti-Ndg antibody (Ndg-B) that recognizes the region between the second G2 domain up to the fourth EGF repeat of Ndg [34]. (B) While NdgΔRod-G3.1 embryos do not show any Ndg staining in embryonic BMs, expression in chordotonal organs is unchanged (arrowheads). (C) NdgΔRod-G3.1 and Ndg2 mutant embryos stained with an anti-Ndg antibody that recognizes an epitope in the Rod domain (Ndg-A; this work) do not show any staining. Scale bars represent 20μm (A-D). (TIF) [file pgen.1007483.s003.tif]

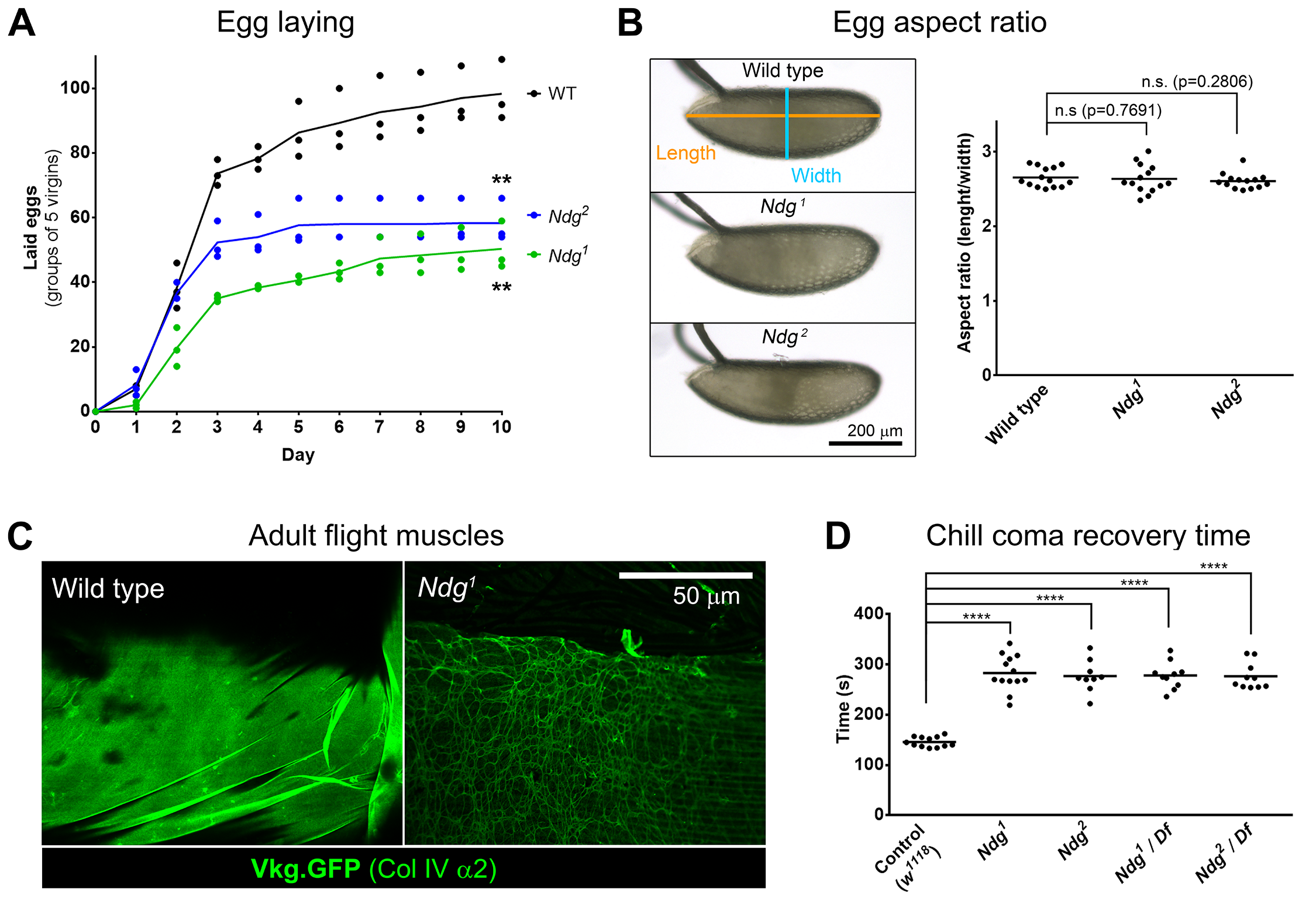

Supplement: S4 Fig — (A) Quantification of eggs laid by wild type (w1118), Ndg1 and Ndg2 virgin females. The curves join mean values of three experiments (individual dots). Differences with the wild type are significant in Kolmogorov-Smirnov tests (**: p<0.01). (B) Images of eggs laid by wild type (w1118), Ndg1 and Ndg2 flies and graph quantifying egg aspect ratio (length/width). Each dot in the graph is a measurement from a single egg. Differences with the wild type were not significant in unpaired two-tailed Student’s t tests. (C) Images of the BM (Vkg.GFP in green) of adult flight muscles, showing the BM is broken in Ndg1 mutants. (D) Quantification of chill coma recovery time in adult female control flies, Ndg1 mutant, Ndg2 mutant, Ndg1/Df(2R)BSC281 and Ndg2/Df(2R)BSC281. Each dot in the graph is a measurement from a single fly. Differences with the wild type were significant in two-tailed t tests with Welch’s correction (****: p<0.0001). (B, D) Horizontal lines represent mean values. (TIF) [file pgen.1007483.s004.tif]

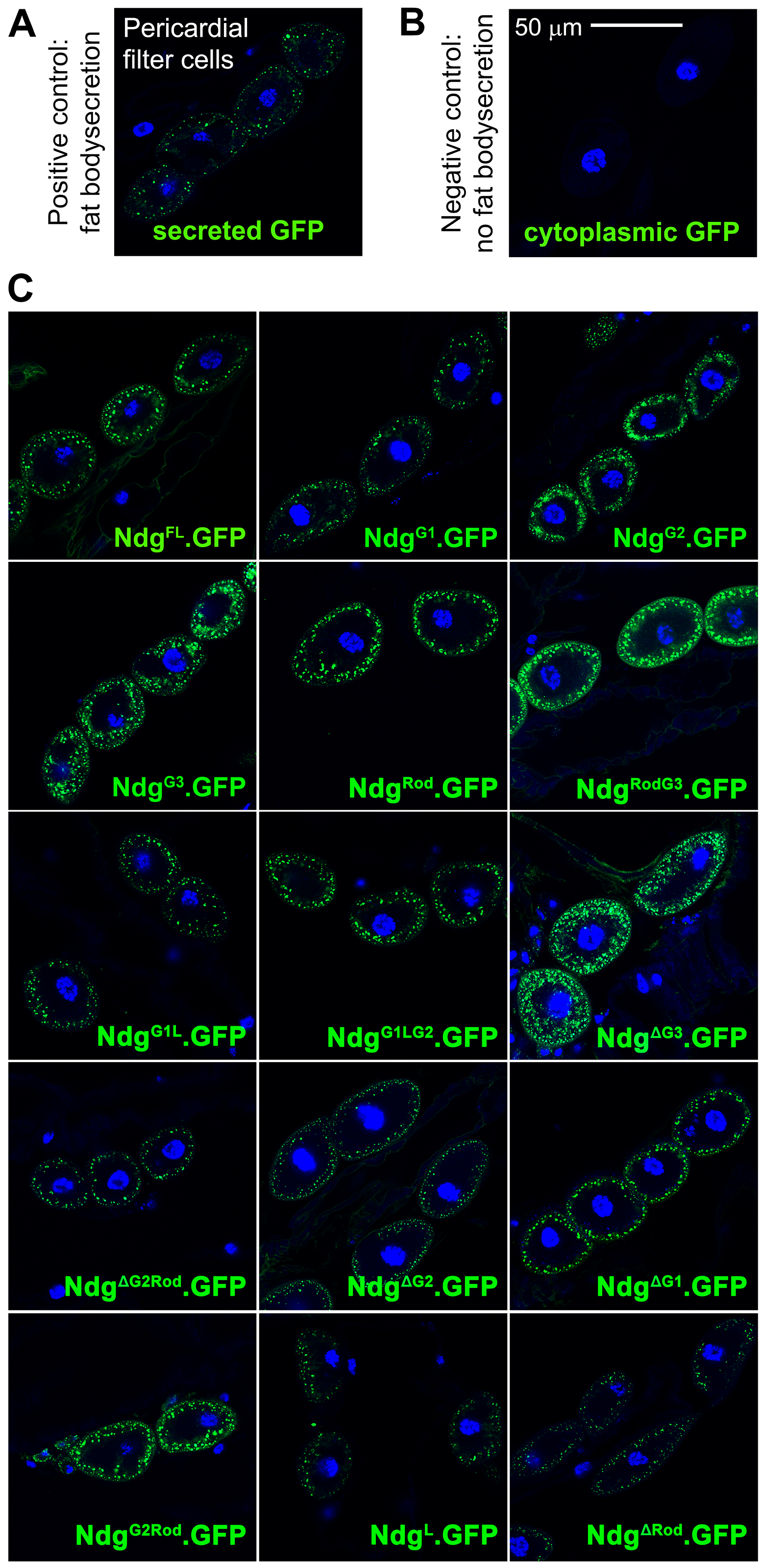

Supplement: S5 Fig — (A) Confocal images showing accumulation of secretion marker secr.GFP (signal peptide of Wg coupled to GFP, green) in pericardial filter cells of Cg>secr.GFP larvae. (B) Pericardial filter cells do not accumulate cytoplasmic GFP expressed in fat body and blood cells (Cg>GFP.S65T). (C) Confocal images of pericardial filter cells showing accumulation of GFP-tagged Ndg variants used in this study (see Fig 5). These variants were expressed in fat body and blood cells under control of Cg-GAL4 and their presence in pericardial cells proves they are secreted. Nuclei stained with DAPI (blue). (TIF) [file pgen.1007483.s005.tif]

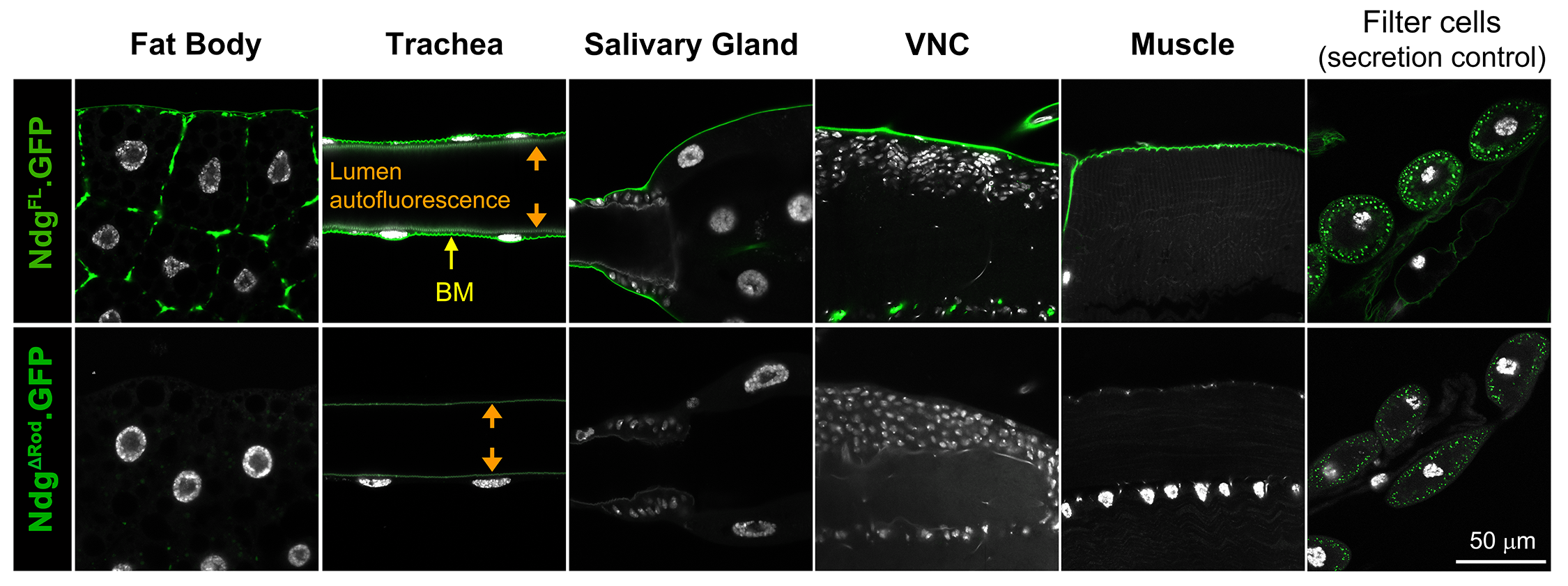

Supplement: S6 Fig — Confocal images of the fat body, trachea, salivary gland, VNC, muscles and filter cells (secretion control) from Cg>NdgFL.GFP (upper panels) and Cg> NdgΔRod.GFP (lower panels) larvae. GFP in green. Nuclei stained with DAPI (white). (TIF) [file pgen.1007483.s006.tif]

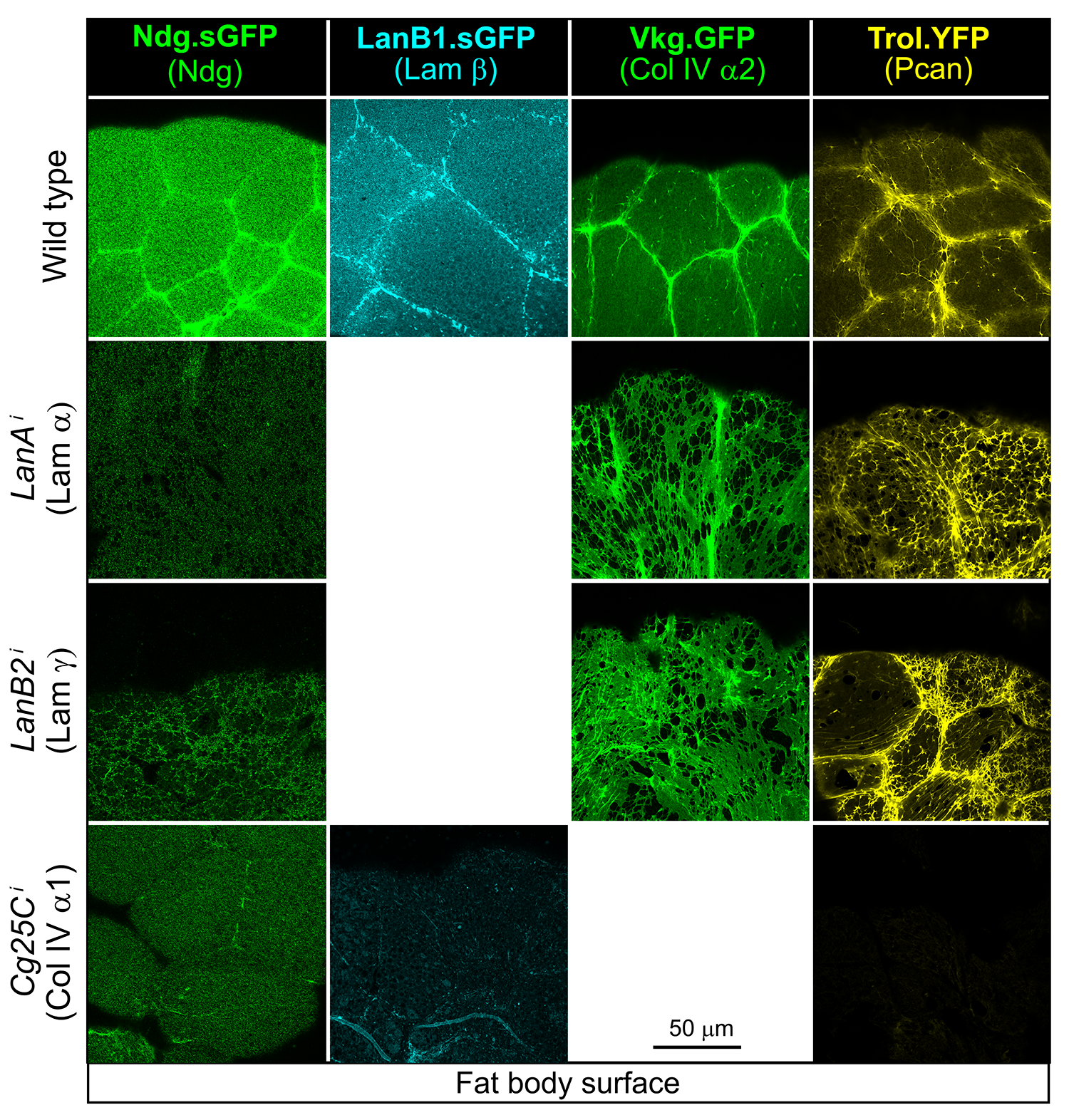

Supplement: S7 Fig — Confocal images of the fat body BM showing localization of Ndg (Ndg.sGFP, green), laminin (LanB1.sGFP, cyan), Collagen IV (Vkg.GFP, green) and Perlecan (Trol.YFP). Images show fat body from wild type larvae (upper panels) and larvae where LanA, LanB2 or Cg25C have been knocked down under control of Cg-GAL4. (TIFF) [file pgen.1007483.s007.tiff]
